# Linking economic growth and international trade taxes in Turkey: A Fourier approach

**DOI:** 10.1016/j.heliyon.2024.e28741

**Published:** 2024-03-27

**Authors:** Alperen Ağca, Onur Uçar, Şükrü Ufuk Uladi̇

**Affiliations:** a Department of Economics, Osmaniye Korkut Ata University, Turkey; b Department of Business, Osmaniye Korkut Ata University, Turkey

**Keywords:** Fourier unit root tests, Fourier VAR, Economic growth, Tax

## Abstract

Revenues from international trade and transactions are crucial for the economic growth and macroeconomic activity of a country. For that reason, taxes from international trade and transactions are used to proxy revenues. Moreover, industrial production index is considered to be proxied for the economic growth. In this study, these variables are used monthly for the period of January 2006 and February 2022 for Turkey. First, different kinds of Fourier based unit root tests including flexibility and fractionality have been used to analyze the data structure. Then, linear VAR and Fourier VAR models have been estimated to reach and analyze the differences of the results of impulse responses between linear and trigonometric models. Finally, linear and Fourier term included Granger Causality tests have been used to question if there would be a difference or not. When the estimations and tests having Fourier structure have been established, the tests considering it have given more different results than linear models. Briefly, it has been shown that policy makers should shift their decisions to an alternative direction.

## 1 Introduction

Value added tax (VAT from now on), which is the most significant revenue resource for the countries all around the world, has been introduced by German entrepreneur Wilhelm von Siemens [1]. VAT contains different sorts of items in its structure. Besides, the tax regulation of countries or decision to determine which items belong to VAT is a controversial topic. While VAT was being imposed by governments such as France on markets, some countries applied VAT on not only domestic trade but also international trade [2]. Although taxes are considered to decrease output level in countries, As is an indirectly imposed VAT, most of the studies indicates that there is a positive relationship between VAT and economic growth by increasingly empowered government revenues [3]. When the central government income items are examined, it is seen that almost all the income from international trade and transactions consists of customs duty and value added tax on imports. Other foreign trade revenues, which are insignificant compared to the revenues obtained from taxes mentioned before, also constitute the revenue item from international trade and transactions. Later on the study, brief explanations will be made about customs duty and VAT on imports.

Customs duty is an indirect tax on the goods included in the customs tariff, even though they enter the customs territory of the Republic of Turkey [4]. Imports have lost their importance with the inclusion of the Value Added Tax (VAT), but in addition to its financial purpose, its easily reflectable nature has made the customs tax one of the important policy tools applied to achieve economic purposes, especially foreign trade policies.

VAT is a general consumption tax that covers all goods and services and covers all economic stages from production to consumption, but, being true to its name, it is a general consumption tax that accepts the added value created in these stages as a base [4]. While the deliveries and services realized within the framework of commercial, agricultural, industrial and self-employment activities constitute two separate groups in the context of transactions subject to VAT, the other group consists of imports of all kinds of goods and services. This is because the principle of country of destination is valid in the taxation of goods or services that are subject to trade in the international arena. For this reason, while the VAT in the imported goods and services is cleared by the exporting countries, it is re-included with the taxation made during import by the importing countries.

## 2 Literature review

As a result of the literature research, it has been determined that the only study for Turkey that specifically examines the relationship between economic growth and taxes on international trade and transactions belongs to Ref. [5]. Using the Toda-Yamamoto Causality Analysis used in their study, the authors concluded that there is no causal relationship between the variables, while they determined the existence of a one-way and temporary relationship from economic growth to tax revenues from international trade and transactions through Frequency Causality Analysis. Apart from the mentioned study, other studies in the literature mainly tested the relationship between economic growth and tax revenues in general terms. However, in order to contribute to the formation of this study and to shed light on other studies to be done, it was thought that it would be appropriate to mention the studies with the aforementioned qualifications.

[6] has examined the relationship between economic growth and tax revenues in Greece for the 1965–2002 periods. The results of the causality tests which are used by the authors in their studies have revealed that there is a causal relationship between economic growth and tax revenues [7]. has investigated the Turkish economy by applying co-integration and Granger causality tests to the data for the period 1975–2006 period. According to the findings obtained from the co-integration test, it has been concluded that economic growth and tax revenues act together, while the findings of the causality test prove the existence of a one-way causality relationship from direct taxes to growth. [8], on the other hand, has taken into account the data between 1980 and 2004 for the Turkish economy in order to examine the relationship between economic growth and direct taxes. Using the Engle-Granger co-integration test for long-term relationships, and error correction model and Granger causality tests for the short-term, the authors have concluded that there is bi-directional causality, but there is no causal relationship between economic growth and indirect taxes.

[9], using regression analysis in the study based on the data of the period 1963–2004 in United States economy, has revealed that high tax rates have a negative effect on economic growth.

[10] has empirically examined the relationship between total tax revenues and economic growth in Pakistan. In this study [10] in which ARDL test has been used for the years 1973–2008, it has been concluded that while the effect of total tax revenues was insignificant in the short term, it had a remarkably negative effect on economic growth in the long term. In the study in which the period of 1950–2009 of the Indian economy was considered by Ref. [11], Johansen cointegration test and VECM have been applied and in the long run the existence of a causality relationship between economic growth and tax revenues have been revealed.

[12], in which it has been examined the effect of economic growth on tax revenues in Malaysia considering the period of 1970–2009, has concluded that the increase in economic growth will increase the amount of tax revenue. The authors have also determined that the stability to be achieved in the economy will have a positive effect on tax revenue and economic growth both in the short and long term [13]. has examined the relationship between economic growth and tax revenues using the panel data method based on the period of 1960–2009 and found that there is a stronger relationship between economic growth and tax revenue in high-income countries compared to low- and middle-income countries [14]. has also revealed the existence of an indirect relationship between economic growth and tax revenues in the study on the 1980–2007 period of the Nigerian economy. The authors, who have reached this conclusion by using the three-stage least squares estimation technique in their analysis, have also determined that the decrease in tax revenues had increased investments and this had been a positive effect on economic growth. [15], in which it is investigated the existence of the relationship between tax revenues and economic growth based on the 1975–2011 period of the Turkish economy, applied Johansen Juselious cointegration and Granger causality tests to find the long- and short-term relationship. According to the results of [15], while there is no relationship between economic growth and tax revenues for the short term, it has been determined that there is a relationship in the long term [16]. has examined the cointegration and causality relationship between economic growth and tax revenues in the study on the Turkish economy. The authors utilizing cointegration and error correction methodology have revealed the existence of a long-term relationship between economic growth and indirect taxes by analyzing the 1998–2011 period with quarterly data sets. In the light of all the findings obtained by the authors from the study, they have concluded that while the revenues from direct taxes are more effective on economic growth in the short run, those from indirect taxes can affect economic growth in the long run.

[17] has examined the effects of taxes on investments and economic growth in Nigeria. Based on the period 1980–2010, the authors have analyzed the data obtained from the Central Bank of Nigeria by using the ordinary least squares method of multiple regression analysis. [17], in which it is determined that there is an inverse relationship between taxation and investment, and thus economic growth, have argued that taxation is an important tool in achieving growth and development goals.

[18] has evaluated the effects of tax administration and tax revenues on economic growth in the study based on the 1990–2012 period of the Nigerian economy. Evaluations in Ref. [18], the authors have benefited from the data obtained by the survey and regression analysis, and as a result, they had deduced that the quality of the tax administration and the amount of tax revenue were effective on economic growth in the mentioned periods. In another study conducted in the same year for the Nigerian economy [19], has revealed that there is a remarkable relationship between economic growth and tax revenues from oil sales profits.

[20] has studied the relationship between economic growth and tax revenues, taking into account the years of 1960–2018 in the Turkish economy. While the author had tested the stationarity of the series with the multiple structural break unit root test, the existence of the cointegration relationship between the series was determined using the multiple structural break cointegration test. The author, who made short-term and long-term analyzes by means of canonical regression analysis, finally had determined the existence of a mutual interaction between economic growth and tax revenues in Turkey in both periods. According to the findings obtained from the study, tax revenues increase with the effect of increasing national income with the growing economy, and this increase accelerates the growth in the economy. Because the study reveals that a 1% increase in tax revenues causes an average of 0.97% increase in national income, and it is concluded that the sum of tax revenues supports economic growth.

In the study [21], it has been conducted a Fourier VAR model by handling grain prices and oil prices. Sharp and smooth transitions are indicated by using TAR method. Moreover, traditional and Fourier term included Granger causality have been established.

In the study based on the Turkish economy for the period of 1980–2015, it has been examined the relationship between economic growth and tax types by using the Granger, Toda-Yamamoto and Breitung-Candelon Frequency Domain causality tests [22]. Proving that there is a long-term relationship between economic growth and total tax revenues, income taxes, indirect taxes and direct taxes, the authors also have revealed the existence of a causal relationship between economic growth and total tax revenues, income tax and direct taxes.

In the study [23] investigating how the change in the composition of tax revenues affects long-term growth, has been analyzed the 1970–2009 period data of high, middle and low income 70 countries. It is found that while increasing consumption and property taxes, reducing income taxes has the effect of increasing long-term growth. In the study [23], it has also been concluded that social security premiums and personal income taxes, which are evaluated within income taxes, tend to have a negative effect on growth.

[24] has specifically investigated the effect of value added tax (VAT) on economic growth, considering the 1985–2018 period of the Turkish economy. The authors, who used the structural break cointegration method in their studies, have revealed the existence of a positive relationship between economic growth and VAT.

[25], aiming to reveal the existence of the relationship between VAT and Special Consumption Tax (SCT) and economic growth in Turkey, based on the years 2006–2019, by means of trend analysis method, shows the rate of increase in the said taxes to the gross domestic product (GDP). By comparing them with the GDP) values, it is come to the conclusion that the annual rate of increase in the taxes in question and the economic growth rates follow each other. From this point of view, the authors deduced that VAT and SCT have income-generating characteristics.

[26] has stated that for an economic growth considering economic freedom and taxpayers’ burden it should be taken account that not only expansionary monetary policies need to be applied but also factors such as property rights, trade liberty and freedom to do business have to be considered.

The literature consisting of the economic growth and international (customs duty) taxes or government revenues getting from international trade is lack of non-linear research method, especially both with respect to trigonometric view and smooth transition. Moreover, there is no Fourier study to analyze the causality between taxes and economic growth for Turkish economy. This paper is supposed to contribute to fill this gap with its empirical framework.

## 3 Data and methodology

To represent the economic growth and the revenues from international trade in Turkey, the industrial production index (gr) and the taxes on international trade and transactions (tax) has been used and gathered from OECD statistics database and Republic of Türkiye Ministry of Treasure and Finance, respectively. The date is monthly and interval of January 2006 and February 2022.

**Figure-1:**
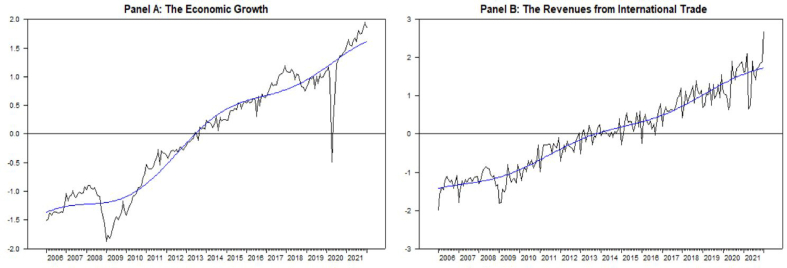
Variables used in the model with fitted values.

First, the data structure has been analyzed and transformed into logarithmic form for both variables (lgr and ltax from now on) (see Figure-1). After the traditional ADF unit root test results indicate stationarity in the first differences of the variables, it has been decided to analyze the Fourier structure of the variables. To maintain that, Fourier based unit root tests, some of which are supposed to give the structural breaks, fractional structures etc., have been used. With respect to ADF and Fourier based unit root tests, the linear VAR and Fourier VAR has been established to analyze and compare the effects of the between each other with impulse responses. Afterwards, the linear Granger causality and the Fourier term included Granger causality tests have been applied.

Although the reduced form of Fourier based models including unit root tests, VAR and causality tests are given below, the proof of the models is not the interest of this study.

Considering the data generator process as,

(1) $$\begin{array}{c} y_{t}=X_{t}^{\prime }\beta +Z_{t}^{\prime }\gamma +r_{t}+\varepsilon_{t}\\ r_{t}=r_{t-1}+u_{t}\text{,} \end{array}$$

[27] has been put the Fourier term into and instead of break point variable $Z_{t}$ which will be equal to ${\left[\sin\;\left(2\pi kt/T\right),\cos\;\left(2\pi kt/T\right)\right]}^{\prime }$ to capture a non-linearity in equation (1), where *k* and *T* denotes the frequency and sample size, respectively. When $Z_{t}$ is considered to be absent, traditional KPSS test would, of course, be as:

(2) $$\tau_{KPSS}=\frac{1}{T^{2}}\frac{\sum_{t=1}^{T}{\hat{S}}_{t}^{2}}{{\hat{\sigma }}^{2}}$$

In equation (2), ${\hat{S}}_{t}=\sum_{j=1}^{t}{\hat{e}}_{j}$ and ${\hat{e}}_{j}$ OLS residuals obtained from regressing $y_{t}$ on $X_{t}$. However, putting the unknown number of breaks into the form will make it need a new function as:

(3) $$\alpha \left(t\right)=\alpha_{0}+\sum_{k=1}^{n}\;a_{k}\;\sin\;\left(\frac{2\pi kt}{T}\right)+\sum_{k=1}^{n}\;b_{k}\;\cos\;\left(\frac{2\pi kt}{T}\right);n<\frac{T}{2}\text{,}$$

Where *n* denotes to numbers of frequencies and *t* denotes trend. Here, they have transformed equation (3) into a single frequency one in equation (4) for get rid of unknown numbers of frequencies:

(4) $$\alpha \left(t\right)≅Z_{t}^{\prime }\gamma =\gamma_{1}\;\sin\;\left(\frac{2\pi kt}{T}\right)+\gamma_{2}\;\cos\;\left(\frac{2\pi kt}{T}\right)$$

Finally, it is modified the KPSS equation using equations (5), (6):

(5) $$y_{t}=\alpha +\gamma_{1}\;\sin\;\left(\frac{2\pi kt}{T}\right)+\gamma_{2}\;\cos\;\left(\frac{2\pi kt}{T}\right)+e_{t}$$

(6) $$y_{t}=\alpha +\beta t+\gamma_{1}\;\sin\;\left(\frac{2\pi kt}{T}\right)+\gamma_{2}\;\cos\;\left(\frac{2\pi kt}{T}\right)+e_{t}$$

The test statistics for Fourier based KPSS is:

(7) $$\tau_{\mu }\left(k\right)\;\text{or}\;\tau_{\tau }\left(k\right)=\frac{1}{T^{2}}\frac{\sum_{t=1}^{T}\;{\tilde{S}}_{t}{\left(k\right)}^{2}}{{\tilde{\sigma }}^{2}}$$

where ${\tilde{S}}_{t}\left(k\right)$ is equal to $\sum_{j=1}^{t}\;{\tilde{e}}_{j}$ and ${\tilde{e}}_{j}$ are residuals for $\tau_{\mu }\left(k\right)$ and $\tau_{\tau }\left(k\right)$ in equation (7).

*F* statistics for the Fourier modified unit root tests are as:

(8) $$F_{i}\left(k\right)=\frac{\left({\text{SSR}}_{0}-{\text{SSR}}_{1}\left(k\right)\right)/2}{{\text{SSR}}_{1}\;\left(k\right)/\left(T-q\right)},i=\mu ,\tau$$

and ${SSR}_{1}$ (k) denotes the SSR from $y_{t}$, *q* is the number of regressors. ${SSR}_{0}$ denotes the SSR from $y_{t}$ without trigonometric terms in equation (8).

When k is unknown as in equation (9),

(9) $$F_{i}\left(\hat{k}\right)=\max_{k}F\left(k\right),i=\mu ,\tau$$

[28] has suggested both ADF and KPSS typed Fourier unit root tests. It has been handled an ESTAR model structure in the study. To save the space the functions of $F_{i}\left(k\right)$, ${FADF}_{i}\left(k\right)$ and $F-t_{{NL}_{i}}\left(k\right)$ are given in equations (10), (11), (12) for both FKPSS and FADF:

(10) $${FADF}_{i}\left(k\right)=\frac{\hat{\delta }}{SE(\hat{\delta )}}$$

(11) $$F_{i}\left(k\right)=\frac{\left({SSR}_{0}-\frac{{SSR}_{1}\left(k\right)}{2}\right)}{\frac{{SSR}_{1}\left(k\right)}{T-q}}$$

(12) $$F-t_{NL}=\frac{\hat{\delta }}{SE\left(\hat{\delta }\right)}$$

The notations are similar with [27] so that the structure of the data which the authors have studied indicates a smooth transition.

[29] has introduced a flexible form of the Fourier based unit root tests except with a better size and a powerful test. The test in Ref. [29] has been using a different variant of the flexible Fourier form of [30]. Besides, the suggested test is reliable whether the data structure is logistic or exponential.

(13) $$F\left(\hat{k}\right)=\frac{\left({SSR}_{0}-\frac{{SSR}_{1}\left(k\right)}{2}\right)}{\frac{{SSR}_{1}\left(k\right)}{T-q}}$$

(14) $$F\left(\hat{k}\right)=\max_{k}F\left(k\right)$$

(15) $$\tau_{LM}=\frac{\hat{\delta }}{SE(\hat{\delta )}}$$

From equation (13), (14), (15), the $F\left(\hat{k}\right)$ and $\tau_{LM}$ functions above, which of the notations such as *T, k, q* and *SSR*s are similar to former unit root tests, are based on the Fourier regressions below:

(16) $$\Delta y_{t}=a+\gamma_{1}\;\sin\left(\frac{2\pi kt}{T}\right)+\gamma_{2}\;\cos\left(\frac{2\pi kt}{T}\right)+\delta y_{t-1}+v_{t}$$

(17) $$\Delta y_{t}=a+\beta t+\gamma_{1}\;\sin\left(\frac{2\pi kt}{T}\right)+\gamma_{2}\;\cos\left(\frac{2\pi kt}{T}\right)+\delta y_{t-1}+v_{t}$$

equations (16), (17) which are the reduced forms of first-differenced regressions. To save place, detrended versions of the equations are not indicated.

A different approach which allows flexible form to be more sensitive and powerful to capture the stationarity or non-linearity of the data structure is the fractional form of unit root test introduced by Ref. [31]. Contributed to Ref. [29] flexible form of Fourier based unit root test is that *k,* the frequency term, could be fractional. In that case, the model suggested only differs from Ref. [29] with respect to interval of the values of *k*. However, this difference makes the model more powerful and better with size. Therefore, fractional frequency flexible Fourier Form (FFFFF) is more sensitive to capture the linearity or non-linearity compared to the integer frequency [31]. The empirical indications are similar to Ref. [29] except the value of $k^{fr}$ is between 0.1 and 2. The grid search method gives a chance to continue to search for frequency value higher than the maximum value of $k_{\max}^{fr}$. Nevertheless, it is unlikely that the fractional frequency value would be higher than 2.

After the unit root tests, the linear and Fourier frequencies included VAR has been established to see the impulse responses from them. Afterwards, linear Granger Causality and Fourier term included one has been analyzed. VAR and FVAR model, which [21] has used in the study, can be shown as:

(18) $$z_{t}=\delta +\sum_{t=1}^{11}\;A_{t}z_{t-t}+e_{t}$$

(19) $$z_{t}=\delta \left(t\right)+\sum_{t=1}^{11}\;A_{t}z_{t-1}+e_{t}$$

where $z_{t}$ represents a function of ltax and lgr, δ represents (2x1) vector of intercepts, $A_{t}$ represents the (2x2) coefficient vector in equations (18), (19). Finally, $e_{t}$ is the orthogonalized vector. Shown as:

(20) $$\left[\begin{array}{c} {e_{t}}^{lgr}\\ {e_{t}}^{ltax} \end{array}\right]=\left[\begin{array}{cc} a_{11} & 0\\ a_{21} & a_{22} \end{array}\right]\left[\begin{array}{c} \varepsilon_{t}^{lgr}\\ \varepsilon_{t}^{ltax} \end{array}\right]$$

where $\varepsilon_{t}$ is the (2x1) vector orthogonal effects of the variables in equation (20). Also, for selecting the lag value, AIC and SIC have been used. Since the data used is monthly, it's allowed to be 12 lags for each equation.

For the FVAR equation, intercept term, $\delta_{i}\left(t\right)$, is included for which *n* values have Fourier frequencies as indicated in equations (21), (22):

(21) $$\delta \left(t\right)={\left[\delta_{1}\left(t\right),\delta_{2}\left(t\right),\delta_{3}\left(t\right),\delta_{4}\left(t\right),\delta_{5}\left(t\right),\ldots \ldots \ldots ,\delta_{n}\left(t\right)\right]}^{\prime }$$

(22) $$\delta_{i}\left(t\right)=a_{i}+b_{t}t+\sum_{k=1}^{n}\;a_{ik}\;\sin\;\left(2\pi kt/T\right)+b_{ik}\;\cos\;\left(2\pi kt/T\right)$$

## 4 Empirical discussion

Both logarithmic variables are stationary in their first differences. Different sort of Fourier unit root tests has been used to see the structure of the data and whether they are consistent of each other or not.

Firstly, KPSS typed Fourier unit root test suggest by Ref. [27] has been used as indicated at the Table-1.

**Table-1:** BEL (2006) fourier KPSS unit root results.

BEL (2006)						
		Intercept		Intercept and Trend		
	*k*	$F_{\mu }$	$\tau_{\mu }$	*k*	$F_{\tau }$	$\tau_{\tau }$
**ltax**	1	121.731	0.396	3	8.829	0.099
**lgr**	1	181.246	0.398	3	41.807	0.069

**Figure-2:**
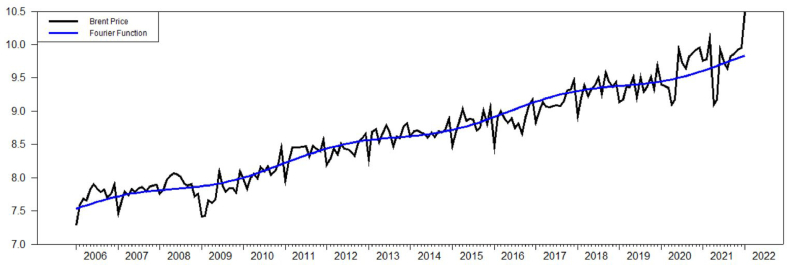
Ltax with intercept term and trend, BEL (2006).

**Figure-3:**
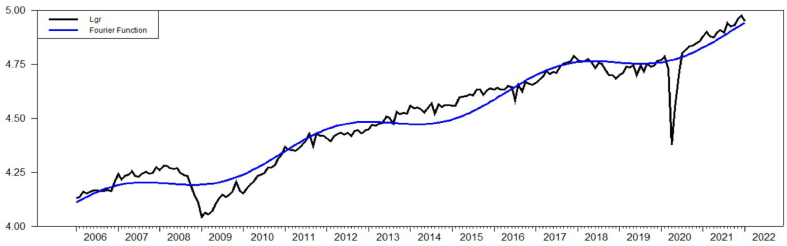
Lgr with intercept term and trend, BEL (2006).

According to Ref. [27], frequency term *k=1* with the intercept term and *k=3* with the intercept term and trend for both lgr and ltax. The value of $F_{\mu }\left(k\right)$ of ltax with intercept term is equal to 121.731 and lgr with intercept term is equal to 181.246 which are way higher than the critical value of [27] which is 4.929 with 5% significance level. The value of $\tau_{\mu }\left(k\right)$ of ltax and lgr with intercept terms are equal to 0.396 and 0.398, respectively. Both values are higher than the critical value of 0.172 with 5% significance level. When looking at the intercept term and trend, $F_{\tau }\left(k\right)$ for ltax and lgr are 8.829 and 41.807 which are again higher than critical value of 4.929. However, looking at $\tau_{\tau }\left(k\right)$ values of ltax and lgr, which are equal to 0.099 and 0.069, they are lower than the critical value of 0.142 with 5% significance level. As a result, both ltax and lgr have Fourier unit root structure with the intercept term and trend as indicated at Figure-2 and Figure-3.

Secondly, Fourier-ADF test which has been developed by Ref. [28] has been used to test the variables. $F_{\mu }\left(k\right)$ values of [28] FADF test are similar to Ref. [27] in both with the intercept term and the intercept term and trend as it can be seen at the Table-1 and Table-2.

**Table-2:** CLL (2010) fourier unit root tests.

CLL FADF (2010)						
		Intercept		Intercept and Trend		
	*k*	$F_{\mu }$	*FADF*_*μ*_	*k*	$F_{\tau }$	FADF_*τ*_
**ltax**	1	121.731	−1.569	3	8.829	−7.051
**lgr**	1	181.246	−1.402	3	41.807	−4.660
**CLL FKSS (2010)**						
		**Intercept**		**Intercept and Trend**		
	***k***	$F_{\mu }$	***F-t***_***NLμ***_	***k***	$F_{\tau }$	***F-t***_***NLτ***_
**ltax**	1	121.731	−0.575	3	8.829	−4.538
**lgr**	1	181.246	−0.228	3	41.807	−5.607

However, μ and τ values of FADF test are far different from Ref. [27] as nominal values. ${FADF}_{\mu }$ value for *k=1* is equal to −1.569 for the variable ltax and −1.402 for the variable lgr which are higher than the critical value of −3.78 for 5% significance level with *T=250*. The results show that the existence of unit root cannot be rejected as the null hypothesis claims. On the contrary of ${FADF}_{\mu }$ values, ${FADF}_{\tau }$ values with the intercept term and trend for *k=3* are −7.051 and −4.660 for ltax and lgr, respectively. Since the critical value is equal to −3.80 for 5% significance level with *T=250*, it can be concluded that the existence of unit root test has to be rejected.

**Figure-4:**
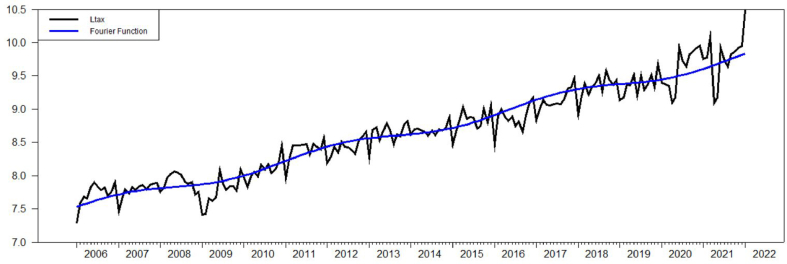
Ltax with intercept term and trend, CLL (2010).

**Figure-5:**
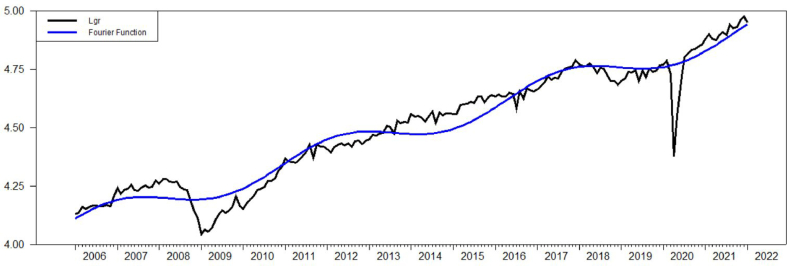
Lgr with intercept term and trend, CLL (2010).

Another Fourier based unit root test suggested by Ref. [28] is Fourier-KSS (FKSS) shows similar results with respect to ${FADF}_{\mu }$ values in both the intercept and the intercept and trend as indicated in Table-1 and Table-2. Moreover, it can be seen that Figure-2, Figure-4 and Figure-3, Figure-5 are exactly the same. However, ${F-t}_{NL\mu }$ value with the intercept term and ${F-t}_{NL\tau }$ value with the intercept and trend which are driven from an exponential smooth transition autoregressive (ESTAR) model indicates that the existence of unit root cannot be rejected for the model with the intercept term but has to be rejected for the model with the intercept term and trend. So that, ${F-t}_{NL\mu }$ value −0.575 with *k=*3 is lower than the critical value of −3.17 for 5% significance level with *T=250* while ${F-t}_{NL\tau }$ value −4.53868 with *k=3* is higher than the critical value of−3.63 for 5% significance level with T=250. Consistent to previous tests, the model indicates a Fourier based structure as it can be seen in Figure-4 and Figure-5.

In the study [29] has established a flexible Fourier unit root test that mentioned before. The empirical results for both the intercept term only and the intercept and trend are more different than the previous tests with respect to nominal values of course. Therefore, the structure contains flexibility. As indicated at Table-3, F(k) values, *k=1,* are equal to 0.855 and 1.137 for ltax and lgr with the intercept term, respectively. These values are lower than the critical values which are calculated for *T=193* in Appendix. Nevertheless, analyzing the $\tau_{LM}$ values, they are higher than the critical value mentioned before. Looking into test with intercept term and trend, although F(k) values, for *k=*2, are still lower than the critical ones, $\tau_{LM}$ values for both variables are lower than the critical value of −4.01 for 5% significance level with *T=193*. This show that the variables are stationary with intercept term and trend.

**Table-3:** EL (2012) flexible frequency fourier unit root test.

EL (2012)						
		Intercept		Intercept and Trend		
	*k*	*F(k)*	*τ*_*LM*_	*k*	*F(k)*	*τ*_*LM*_
**ltax**	1	0.855	−1.491	2	3.897	−7.039
**lgr**	1	1.137	−0.989	2	4.237	−4.611

Based on [31] fractional frequency form of a flexible Fourier unit root test, handling ltax, for both intercepts only and the intercept term and trend, $F^{fr}$ values, *k=0.1,* for ltax variable are equal to 22.970 and 2.515. The value for intercept only is higher than the critical value of 9.85 for %5 significance level with *T=200* while the other is not. $\tau_{DF}^{fr}$ test statistics for ltax with both intercepts only and the intercept term and trend are −6.777 and −6.738, respectively that are lower than the critical value of −3.90 for %5 significance level with *T=200* as it is stated in Table-4. On the other side of the page, $F^{fr}$ and $\tau_{DF}^{fr}$ the variable lgr is not statistically significant with respect to all point of view. One can only claim that further studies based on the fractional frequency forms of non-linear econometric estimation models have to consider FFFFF unit root test while testing the data structure and benefit from it [31]. The form that taking account into fractionality seems to be more powerful to analyze the nonlinearity. However, this very study will cover only VAR and FVAR Models and Granger Causality between the variables with and without including only Fourier terms.

**Table-4:** Omay (2015) FFFFF unit root test.

Omay (2015)						
		Intercept		Intercept and Trend		
	*k*	$F^{fr}$	$\tau_{DF}^{fr}$	*k*	$F^{fr}$	$\tau_{DF}^{fr}$
**ltax**	0.1	22.970	−6.777	0.1	2.515	−6.738
**lgr**	0.2	7.044	−3.838	0.1	0.436	−3.879

**Figure-6:**
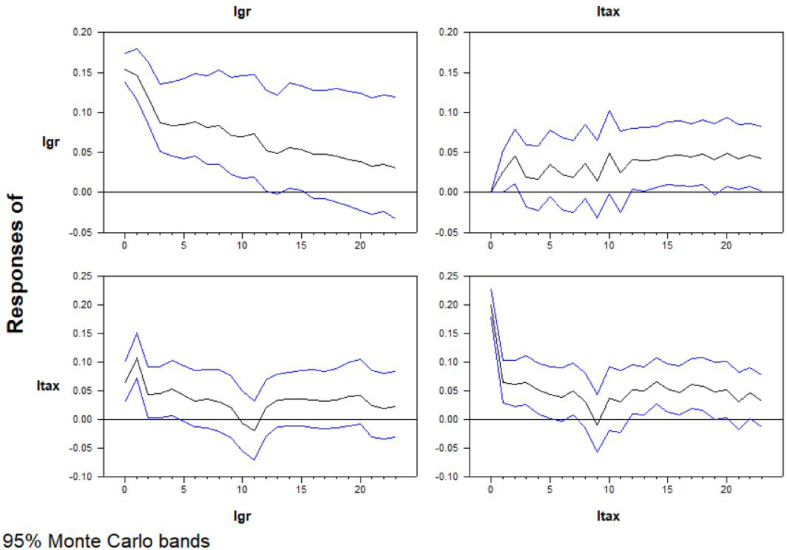
Impulse reponses of VAR

Since the data structure has been analyzed, the linear and Fourier frequency included VAR impulse response results can be evaluated. VAR model system estimation is both significant for ltax and lgr. Monte Carlo simulation has been made with 2000 draws and 24 steps. As it can be seen in Figure-6, a change in the economic growth decreases the revenues from international trade in the long run. However, one can see that there is a clear peak in the first month which might be captured by causality tests. On the other hand, revenues from international trade causes a significant increase in the economic growth.

**Figure-7:**
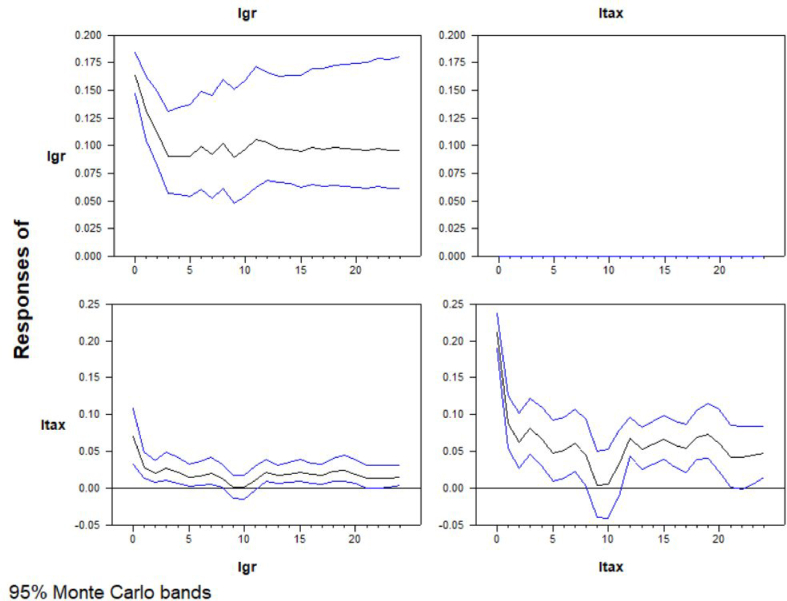
Impulse reponses of FVAR

For *n=*2, *k=*1 and lags allowed to 12, system estimation results in significant in both variables. As it is shown in Figure-1, the fitted values appear to be shifting gradually. In impulse responses of FVAR model estimation in Figure-7, the effect of economic growth seems to be decreasing the revenues from international trade. There is a crucial decrease through the first 2 months. Afterwards, the series are relatively stable. On contrast of linear VAR results in FVAR model there is no response of the economic growth to the revenues from international trade.

According to linear Granger Causality test results, the null hypothesis that revenues from international trade causes the economic growth can be rejected. So that, the *p-value* for the hypothesis can be seen in Table-5 in parentheses. On the other hand, the null hypothesis that the economic growth causes the revenues from international trade cannot be rejected. However, when the Fourier term included in the causality tests, both null hypothesizes cannot be rejected.

**Table-5:** Causality tests.

	Linear Model	Fourier Model
	*Results*	
ltax→lgr	insignificant	**significant**
*F-statistics*	1.569	**2.189**
*Prob*	(0.105)	**(0.015)**
lgr→ltax	**significant**	**significant**
*F-statistics*	**2.908**	**4.048**
*Prob*	**(0.001)**	**(0.001)**

Bold ones indicate 5% significance.

As in the Table-5, both variables granger causes each other in Fourier model. In order to save space, regression computed including seasonal variables for *i=112* and trigonometric functions for *k=*1 has not been written. All in all, it is found that when the Fourier structure is taking into consideration the results differ from the linear model.

## 5 Conclusion

As a crucial aspect of macroeconomic activity, the economic growth of a country has to be handled not only with respect to monetary policies but also with the fiscal policies. Surely, one of the most important components of fiscal policies are taxes which provide significant amount of government revenues. Therefore, taxes from international trade and transactions have been used as a proxy of the revenues. Also, the industrial production index has been proxied for the economic growth in Turkey. Although, mainstream economic schools state that the changes in the amount of the taxes will directly or indirectly affect the economic growth, many studies in the literature predict a growing economy could affect the taxes because of the multiplier effects or the velocity of the financial system. This brings the question whether the different econometric methodologies could indicate different results or not. For this reason, the data structure for both taxes and economic growth has been analyzed by considering the Fourier based tests. First, the Fourier term included unit root tests some which allows the flexibility and/or the fractionality have been used alternative to traditional unit root tests. Secondly, linear VAR model and FVAR model taking into account trigonometric form have been compared to each other with respect to impulse responses. It has been showed that there is a bilateral effect for the economic growth and the revenues from trade and transactions in the linear model for Turkey. However, the same effect is only one way through in the Fourier form such as there is a response of the revenues to a change in the economic growth. Finally, Granger Causality tests have been applied by considering both linear and Fourier structure. While only the economic growth was Granger causing to revenues in linear model, it had been found that there was a bilateral Granger causality in the Fourier term included model. Bidirectional causality between taxes and income indicates that the option for the policy makers can be multitasked. Therefore, they have to decide whether to empower the size or volume of the taxes or not to ensure that the economic growth would not be overwhelmed by increasing taxes, vice versa. As a result, researchers and policy makers should consider not only the stationarity and linear models in their studies but also the Fourier models based on trigonometric form of both data structure and model estimation. Since linear models might not result causality between variables such as tax and economic growth, policy implications based on traditional linear models could be misleading. Therefore, further studies about public finance and economic policy should be include Fourier forms. Policy makers have to be more cautious while applying the tax policies due to the bidirectional causality Until today policy makers have generally implemented policies considering linear models.

In summary, if policymakers only consider and implement linear models in making decisions, they will overlook the mutual causality relationship between growth and taxes. To address the issue arising from this situation, it would be more appropriate for policymakers to also consider trigonometric models in analyzing the relationship between growth and taxes.

## Data availability statement

Sharing research data helps other researchers evaluate your findings, build on your work and to increase trust in your article. We encourage all our authors to make as much of their data publicly available as reasonably possible. Please note that your response to the following questions regarding the public data availability and the reasons for potentially not making data available will be available alongside your article upon publication.

## CRediT authorship contribution statement

**Alperen Ağca:** Writing – review & editing, Writing – original draft, Software, Methodology, Formal analysis, Data curation. **Onur Uçar:** Writing – review & editing, Writing – original draft, Visualization, Resources, Investigation, Conceptualization. **Şükrü Ufuk Uladi̇:** Writing – review & editing, Writing – original draft, Visualization, Methodology, Formal analysis.

## Declaration of competing interest

The authors declare that they have no known competing financial interests or personal relationships that could have appeared to influence the work reported in this paper.

## Figures and Tables

**Table:** 

k	T=100			T=193			T=500		
	1%	5%	10%	1%	5%	10%	1%	5%	10%
**Panel a**: **Critical values of**$\tau_{\mathrm{LM}}$									
1	−4.66	−4.10	−3.81	−4.62	−4.09	−3.81	−4.57	−4.02	−3.76
2	−4.24	−3.58	−3.24	−4.22	−3.54	−3.20	−4.14	−3.56	−3.22
3	−3.99	−3.32	−2.96	−3.97	−3.32	−2.98	−3.95	−3.30	−2.99
4	−3.82	−3.18	−2.86	−3.84	−3.20	−2.86	−3.81	−3.19	−2.88
5	−3.73	−3.14	−2.81	−3.77	−3.13	−2.82	−3.77	−3.12	−2.83
**Panel b: Critical values of**F(k)									
1	6.96	8.48	11.64	6.98	8.22	11.23	6.92	8.22	10.88
2	3.68	4.85	7.62	3.72	4.86	7.38	3.74	4.84	7.43
3	2.96	3.89	6.19	2.91	3.84	5.99	2.91	3.80	6.01
4	2.58	3.32	5.29	2.63	3.39	5.29	2.66	3.44	5.30
5	2.49	3.33	5.29	2.48	3.26	4.97	2.49	3.29	5.08

**Table:** 

k	T=100			T=193			T=500		
	1%	5%	10%	1%	5%	10%	1%	5%	10%
**Panel a**: **Critical values of**$\tau_{\text{LM}}$									
1	−4.94	−4.36	−4.04	−4.91	−4.32	−4.02	−4.82	−4.29	−3.99
2	−4.68	−4.05	−3.71	−4.60	−4.01	−3.67	−4.54	−3.98	−3.68
3	−4.41	−3.80	−3.45	−4.38	−3.77	−3.45	−4.36	−3.74	−3.40
4	−4.26	−3.64	−3.29	−4.24	−3.63	−3.31	−4.24	−3.61	−3.29
5	−4.21	−3.57	−3.23	−4.15	−3.56	−3.24	−4.17	−3.52	−3.22
**Panel b: Critical values of**F(k)									
1	6.99	8.51	11.81	6.96	8.34	11.40	6.90	8.22	11.10
2	4.59	5.89	8.90	4.52	5.82	8.72	4.52	5.76	8.48
3	3.37	4.48	6.85	3.37	4.33	6.76	3.35	4.23	6.63
4	2.82	3.62	5.78	2.85	3.67	5.82	2.91	3.80	5.81
5	2.67	3.56	5.60	2.67	3.53	5.30	2.66	3.52	5.44

The critical values have been calculated with 10000 iterations.
